# An mHealth Workplace-Based “Sit Less, Move More” Program: Impact on Employees’ Sedentary and Physical Activity Patterns at Work and Away from Work

**DOI:** 10.3390/ijerph17238844

**Published:** 2020-11-28

**Authors:** Judit Bort-Roig, Emilia Chirveches-Pérez, Maria Giné-Garriga, Lydia Navarro-Blasco, Roser Bausà-Peris, Pedro Iturrioz-Rosell, Angel M. González-Suárez, Iván Martínez-Lemos, Emma Puigoriol-Juvanteny, Kieran Dowd, Anna Puig-Ribera

**Affiliations:** 1 Sport and Physical Activity Research Group, Centre for Health and Social Care Research, University of Vic-Central University of Catalonia, 08500 Barcelona, Spain; annam.puig@uvic.cat; 2 Research Group on Methodology, Methods, Models and Outcomes of Health and Social Sciences (M3O), Centre for Health and Social Care Research, University of Vic-Central University of Catalonia, 08500 Barcelona, Spain; emilia.chirveches@uvic.cat; 3 Department of Physical Activity and Sport Sciences, Faculty of Psychology, Education and Sport Sciences (FPCEE) Blanquerna, Ramon Llull University, 08022 Barcelona, Spain; mariagg@blanquerna.url.edu; 4 Department of Physiotherapy, Faculty of Health Sciences Blanquerna, Ramon Llull University, 08025 Barcelona, Spain; 5 Occupational Health Service, Hospital de la Santa Creu i Sant Pau, 08041 Barcelona, Spain; LNavarro@santpau.cat (L.N.-B.); RBausa@santpau.cat (R.B.-P.); 6 Unidad Docente Pluridisciplinar de Atención Familiar y Comunitaria, Hospital Universitario de Donostia, 20014 Donostia-San Sebastián, Spain; pedro.iturriozrosell@osakidetza.eus; 7 Department of Physical Education and Sport, University of the Basque Country, 01007 Vitoria-Gasteiz, Spain; angelmanuel.gonzalez@ehu.eus; 8 Well-Move Research Group (HI-23), Faculty of Educational Sciences and Sports, University of Vigo, 36005 Pontevedra, Spain; ivanmartinez@uvigo.es; 9 Tissue Repair and Regeneration Laboratory (TR2Lab), Faculty of Sciences and Technology, University of Vic-Central University of Catalonia, 08500 Vic, Barcelona, Spain; emma.puigoriol@uvic.cat; 10 Epidemiology Unit, University Hospital of Vic-Vic Hospital Consortium (HUV-CHV), 08500 Barcelona, Spain; 11 Department of Sport and Health Sciences, Athlone Institute of Technology, N37 HD68 Athlone, Co. Westmeath, Ireland; kdowd@ait.ie

**Keywords:** mHealth, occupational health, workplace, sedentary behaviour, sitting

## Abstract

Background: Most workplace interventions that aim to reduce sedentary behaviour have 38 focused on employees’ sedentary patterns at-work but less have focused on understanding the 39 impact beyond working time. The aim of this study was to evaluate the impact of a 13-week m-40 health workplace-based ‘sit less, move more’ intervention (Walk@WorkApp; W@W-App) on 41 physical activity (PA) and sitting in desk-based employees at-work and away from work. Methods: Participants (*n* = 141) were assigned by hospital to an intervention group (IG; used the W@W-App; *n* = 90) or an active comparison group (A-CG; monitored occupational activity; *n* = 51). The W@W-App, installed on the participants´ own smartphones, provided real-time feedback for occupational sitting, standing, and stepping, and gave access to automated strategies to sit less and move more at work. Changes between groups were assessed for total sitting time, sedentary bouts and breaks, and light and moderate-to-vigorous PA (activPAL3TM; min/day) between the baseline and after program completion. Results: Compared to the A-CG, employees that used the W@W-App program increased their number of daily breaks and the time spent on short sedentary bouts (<20 min, *p* = 0.047) during weekends. Changes in shortest sedentary bouts (5–10 min) during weekends were also statistically significant (*p* < 0.05). No changes in workday PA or sitting were observed. Conclusion: Desk-based employees seemed to transfer the W@W-App program knowledge outside of work. Evaluating the impact of workplace (mHealth-based or not) interventions at work but also away from work would provide a better understating of the impact of such interventions.

## 1. Introduction

In adults, prolonged sedentary behaviour [1] has been associated with a broad range of health consequences, including unhealthy cardio-metabolic biomarkers [2] and a lower physical health-related quality of life [3]. While sitting for an extra hour per day increases the risk for developing type 2 diabetes and metabolic syndrome by 22% and 39%, respectively [4], evidence from a meta-analysis indicated that performing at least 60 min of moderate-to-vigorous physical activity (MVPA) per day can attenuate the hazards associated with excessive sitting time [5]. With frequent interruptions of sitting also adding extra beneficial cardio-metabolic associations [2], leveraging the time-inverse relationship between sedentary and active behaviours can lead to important benefits for public health [6].

Desk-based jobs are major contributors to peoples´ sedentary and inactive patterns [7,8], with office employees spending between 70% and 80% of their working day sitting [9,10]. In a context where occupational sedentary patterns are highly prevalent and associated with an increased risk for chronic diseases and premature mortality [11], searching for effective interventions that target occupational sitting time reductions is fundamental to tackle current public health challenges [12].

Mobile phones have the potential to increase employees´ awareness and empowerment towards changing occupational sedentary and physical activity (PA) patterns [13,14]. Mobile phones are a widely accessible tool that can be used to self-monitor behaviours, while they also have the capability to deliver real-time feedback on PA and sedentary patterns while at work [15]. However, data on the effectiveness of mobile phone interventions (mHealth) for promoting workplace PA and reducing sedentary behaviour is scarce [15]. Even less is known about the impact workplace “sit less, move more” mHealth interventions have on “off-work” sedentary and PA patterns [16,17]. This is particularly important, as preliminary evidence has suggested there may be a “compensation effect” for workplace behaviour interventions. Thus, increases in occupational standing time, PA, and reductions in occupational sedentary time may result in adverse alterations of such behaviours outside working hours [17].

With most evidence focusing on the impact workplace interventions (not mHealth-based) have on occupational sedentary patterns [18], this study evaluated the effectiveness of a Walk@Work app (W@W-App), an mHealth-based workplace “sit less, move more” intervention, on changing both occupational and non-occupational activity behaviours during work days and non-work days in desk-based employees.

## 2. Materials and Methods

A convenience sample of four Spanish hospitals were randomly assigned to an intervention group that implemented the W@W-App (intervention group (IG), *n* = 2) or an active comparison group (A-CG, *n* = 2), made up from their administrative staff. The Occupational Health Services from each hospital contacted all their administrative staff (*n* = 321) and invited them to participate in the study. Two hundred and fifteen employees expressed interest in participating in this study. Employees were considered eligible if they had access to a mobile phone with an Android version >4.0, had no physical or health problems that limited their ability to stand for bouts of at least 10 min, and had no planned absence from work for more than one week over the next two months. Finally, one hundred and forty-one participants were recruited and assigned to the IG (*n* = 90) or A-CG (*n* = 51), according to the hospital they belonged to. Reasons for non-inclusion consisted of not being interested, being very active outside work, or not wishing to use their personal mobile phones. All the participants were blinded to group allocation and provided written informed consent. The study was approved by the ethics committees of each hospital (Hospital Universitario de Donostia, PI2014052; Hospital de Vigo, 2014/248; Hospital de Vic, 2013845/PR75; Hospital de la Santa Creu i Sant Pau, EC/14/149/4013).

**Figure 1:**
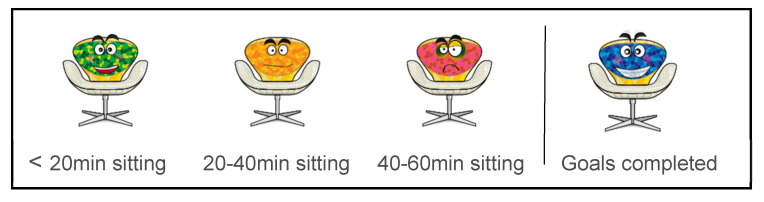
Chair feedback displayed on the Walk@Work app (W@W-App).

The W@W-App was designed to accurately self-monitor occupational sitting, standing, and stepping. The app’s validity and characteristics have been reported elsewhere [19]. The complete W@W-App was installed on the IG participants´ own mobile phones for 13 weeks. The app displayed the employee’s occupational activity in real-time by using a time counter for sitting, standing, and stepping, as well as an emoji of an animated chair. According to the time spent in sitting bouts, the chair changed (see Figure 1) from a happy green chair (<20 min) to an uncomfortable yellow chair (20–40 min), and then to an upset purple chair (40–60 min). When sitting time was prolonged for more than one hour, a vibration feature of the mobile phone was activated. At the end of the working day, the mobile application sent the data to the web server and it returned a daily summary message with the support of a chair image. Participants could see the chair that most represented their working sitting bouts (green, yellow, or purple). Participants from the IG also had access to automated strategies to sit less and move more at work along an 8-week ramping phase and a 4-week maintenance phase that has been described in detail elsewhere [20,21]. Fortnight and weekly messages informed the participants about the strategies and goals at the ramping and maintenance phases, respectively. Additionally, weekly motivational messages reported the progress made with an excited blue chair (see Figure 1) appearing when the goals were completed. For participants in the A-CG, a partial W@W-App was installed onto their mobile phone. This included the self-monitoring features but did not provide feedback on the strategies or goals to change their sedentary behaviour during working hours.

Researchers also supplied an ad-hoc pouch (W@W-App pouch) due to the requirements of hospital staff to wear specific gowns/scrubs at work and not being able to hold the phone in their pockets. The W@W-App pouch allowed participants to place the mobile phone at the right-hand side of their waist. This location has been deemed the most appropriate position to avoid postural measurement errors [19]. The pouch let participants use their own mobile phone (e.g., texting, calling, or internet searching) while also monitoring their activity during working hours. More information on the mobile phone position provided by the W@W-App pouch is described elsewhere [19].

The activPAL3TM (PAL Technologies Ltd., Glasgow, UK) was used to measure and quantify the PA and sedentary behaviours of the desk-based employees. This device has already been demonstrated as a valid measure to quantify body posture and activity patterns during free-living conditions [22,23,24,25]. The device was attached to the participants’ right thigh using a flexible nitrile sleeve and a transparent film (10 × 10 cm of hypoallergenic Tegaderm™ Foam Adhesive Dressing). The waterproof dressing of the activPAL3TM allowed participants to wear the monitor continuously for 24 h per day for 7 complete days at baseline and following 12 weeks of intervention. Participants received additional dressings and instructions on how to reattach the device if needed. Additionally, participants were asked to record their daily wake-up time, bedtime, working hours, and any monitor removal time.

Data were processed using activPAL Professional Software™ (version 7.2.32), Microsoft Excel 2010 (Redmond, WA, USA), and MATLAB v8.4 (MathWorks^®^, Natick, MA, USA), following previously published protocols [22]. From the activPAL3TM software output, the following outcomes were determined: total sitting, standing, and stepping time; total number of sitting bouts; total amount of time spent in sitting bouts; total number of sitting bouts; and time spent on sitting bouts of a different duration (<5 min, 5–10 min, 10–20 min, <20 min, >20 min, 20–30 min, 30–40 min, <40 min, >40 min, 40–60 min, >60 min, and >90 min). Additionally, total time spent in light intensity and moderate-to-vigorous PA was determined by using previously validated count-to-activity thresholds [26]. Overall, the outcomes were reported as averages of the total week, workdays (working and non-working hours), and weekend days. Working and non-working times were established by using the participants’ daily records.

A mean comparison of the repeated measures assessed the changes between groups in the main outcomes for PA and sedentary behaviours between the baseline and after program completion. The magnitude of difference after 13 weeks between the groups was used to identify the intervention effects. Statistical analyses were performed using IBM SPSS Statistics v.26 (SPSS, Inc., Chicago, IL, USA). Significance was set at *p* < 0.05.

## 3. Results

**Figure 2:**
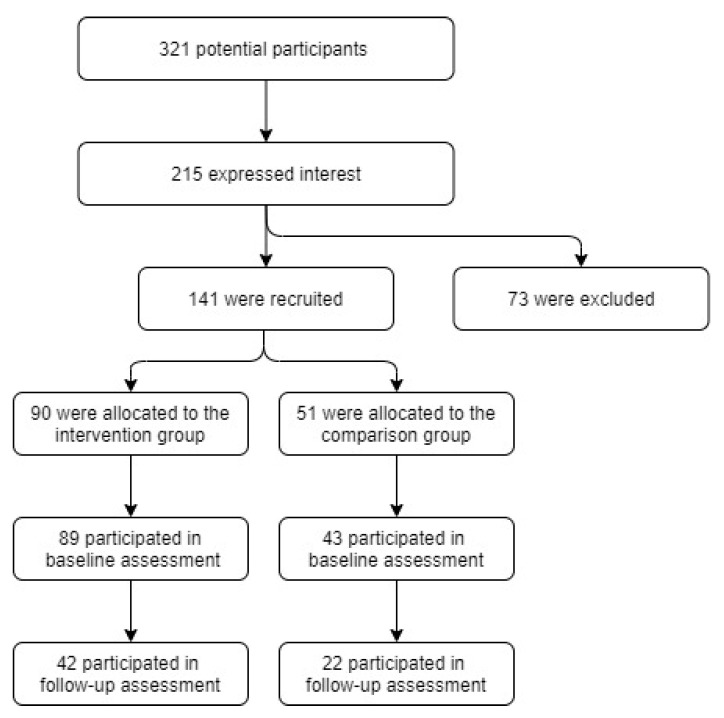
Flow diagram of the study design.

The hospital leaders invited 321 potential participants, with 215 expressing interest in the study. Of these, 73 were excluded due to not having mobile phones with an Android version >4.0 or due to having iPhones. A total sample of 141 administrative staff were recruited (45 ± 9 years; 82% female): 90 were assigned to the IG (W@W-App) and 51 to the A-CG (monitored occupational activity). One hundred and thirty-two participants completed the baseline assessments (IG = 89 and A-CG = 43) while the follow-up assessments were enrolled by 64 participants (IG = 42; and A-CG = 22). The program drop-out was 47% and 51% for the intervention and comparison groups, respectively (see Figure 2), mainly due to battery-life issues.

For both groups combined, the monitored being awake time at baseline was 17.0 ± 0.9 h per day on workdays and 15.4 ± 1.4 h per day on weekends. The mean monitored time at the workplace was 7.5 ± 2.3 h per day and 9.5 ± 2.6 h per day including outside work.

### 3.1. Office Employees’ Sedentary Patterns and Standing at Work, Outside Work, and Weekends

On workdays, employees spent on average 10.0 h sitting per day, of which 4.8 h were at work (60%) and 5.2 h were outside work. Sitting bouts longer than 20 min represented 55% of the total sitting time. During work time, a total of 52% of occupational sitting time was accumulated in short sitting bouts (<20 min). On weekend days, sitting time accounted for 8.6 h/day, of which sitting bouts longer than 20 min represented 68% of total sitting time. Standing time accounted for approximately 5 h on both workdays and weekend days, with only 1.9 h standing accumulated during working hours. A more detailed description of the office workers’ sedentary patterns is presented in Table 1, and the group differences are described in Supplementary Materials S1.

### 3.2. Office Employees’ PA Patterns at Work, Outside Work and Weekends

Employees accumulated approximately 2 h of movement (stepping time) per day, with no differences in the amount of stepping time accumulated on workdays or weekend days (2.03 ± 0.50 and 1.89 ± 0.78, respectively). The PA intensity slightly varied between workdays and weekends, with more time spent on MVPA (+13 min) during workdays than weekends (47 min vs. 34 min, respectively) and more time spent on light intensity PA (+5 min) during weekends than workdays (80 min vs. 75 min, respectively). On workdays, daily stepping time was mainly accumulated outside work (1.31 h or 78 min) compared to the 0.71 h (42 min) accumulated while working. Light intensity PA was mainly accumulated outside work (50 min) when compared to work time (25 min). Similarly, MVPA was performed for an average of 12 min extra outside work (30 min) compared to working hours (18 min). A more detailed description of office workers’ PA is presented in Table 1, and the group differences are described in Supplementary Materials S1.

### 3.3. Effect of the Walk@Work App mHealth Intervention on the Office Employees’ Sedentary and PA Patterns at Work, Outside Work, and on Weekends

Table 2 describes how the outcomes changed due to the mHealth intervention. Compared to the baseline, the W@W-App participants moved more at work (+46 min stepping, *p* = 0.039), reporting small but statistically significant changes in occupational light intensity activity (+2.4 min, *p* = 0.031). Changes in MVPA were only reported during weekends (+10 min, 0.002), while no changes were found in the PA patterns outside work during workdays. Changes in standing time were not observed. At post intervention, sitting time remained consistent but changes were observed in the way employees accumulated their sitting time during workdays non-working hours, performing more sitting breaks every 20 to 30 min (*p* = 0.037) and spending more time in shorter sedentary bouts from 20 to 30 min (*p* = 0.040), what indicates a change on their sitting patterns, breaking the time sitting more often. However, no significant differences between groups were identified for these outcomes.

Differences between groups represented the intervention effects, which were only identified during weekends for sedentary bouts and breaks from sitting time. Compared to the A-CG, employees that enrolled the W@W-App program increased the number of daily breaks and the time spent on short sedentary bouts (<20 min, *p* = 0.047) only during weekends. Changes in short sedentary breaks and time spent on bouts between 5 and 10 min at weekends were also reported to be statistically significant (*p* = 0.042 and 0.045, respectively).

## 4. Discussion

This study evaluated the impact of an mHealth workplace program on PA and sedentary behaviours quantified using a device-based measure in desk-based employees, both at work, outside work, and during weekends. In comparison to the A-CG, the W@W-App intervention resulted in a greater number of sitting breaks and more time spent in sedentary bouts of a shorter duration on weekends, but not during working or non-working hours on workdays. Although our findings did not reveal reductions in the total or occupational sitting time, changes in the distribution of sedentary bouts were shown on weekends when employees have more flexibility to choose what to do [21]. The W@W-App program was designed to enhance active jobs, but instead changes were observed in non-occupational-based settings (i.e., during weekends). Because frequent interruptions of sitting time are associated with enhanced cardio-metabolic biomarkers [2], improving sedentary patterns outside work is beneficial for public health.

Other “sit less” interventions using sit-stand desks have significantly reduced sedentary time and increased light activity levels during working hours, but evidence suggests that these changes tend to be compensated for by reduced activity and increasing sitting outside working hours [17]. The hypothesis that as PA increases in one domain (i.e., at work) a compensatory change happens in another domain (i.e., outside work) [27] was not supported by our findings. This may be explained because interventions using sit-stand desks mainly focus on changing the workplace built environment, while the W@W-App program focuses on educational and motivational strategies to help employees reduce their sedentary patterns, which can also be transferred to outside work patterns.

In this context, current PA guidelines for adults propose the accumulation of 300 min/week in leisure or transport PA for those working in desk-based jobs [28], assuming that the PA levels at work might be more difficult to change than other domains and recommending that employees who sit for most of their working time should be more active outside work [29]. Our findings indicate that employees transferred the program knowledge outside of work, performing more sitting breaks and spending more time on short bouts during weekends, which indicates the need to evaluate occupational “sit less, move more” interventions not only on changes for sitting and PA behaviours during work hours but also outside work.

Other workplace interventions have focused on walking strategies, showing mixed results on their effectiveness for reducing occupational sitting time [20,30,31]. According to a recent systematic review [15], adding mHealth interventions that employ wearable activity monitors alone or together with mobile phone applications is an innovative strategy that could enhance the effectiveness of workplace programs aiming to reverse prolonged sitting time. Using mobile phone applications and wearables are strong potential mechanisms to change sedentary behaviour at a low-cost [13], with our study indicating they can also have an impact on sedentary and PA patterns outside work.

### Strengths and Limitations

To our knowledge, this is the first study to evaluate the impact of an mHealth workplace-based program not only during work time but also during non-work hours in the workdays and over weekends. This has contributed to the limited number of workplace “sit less, move more” interventions that have studied the impact of such interventions on “out of work” PA and sedentary patterns.

In addition, the Walk@Work app has unique features, including (i) a combined mobile phone in-built accelerometer that provides real-time feedback on employees sitting, standing, and moving; (ii) combined individual and organizational strategies designed to encourage incidental movement and short and longer walks at work; and (iii) utilised motivational and persuasive text messages to target changes in undesirable occupational sedentary behaviours.

Several factors might have influenced the lack of effects identified in the W@W-App IG on occupational sedentary and PA patterns when compared to the comparison group. First, both groups had access to a mobile phone application (the IG had access to the full W@W-App while the AC-G had access to a partial W@W-App with only the self-monitoring features opened). To best determine the impact of this mHealth intervention on occupational PA and sedentary behaviour, the use of a third group with no access to any self-monitoring features or the use of a control group with no access at all to the mHealth program would have been required. However, the Occupational Health Services from hospitals believed it was more ethical to offer the mHealth program to all participants with availability of limited features. Nonetheless, this study indicated that having access to valid and accurate occupational sitting measures with no training program also seemed to influence the occupational sedentary and PA patterns. Second, employees were provided with an ad-hoc pouch to place the mobile phone at the right-hand side of their waist. Although this location has been identified as the best position to avoid postural measurement errors, the feasibility of this tool may be limited. Third, the high rate of participants’ dropouts, mainly due to battery issues with the app, limited the results of the current study. Alternatively, ongoing research is exploring the integration of a low-cost commercially available sensor (MetaWearC; MbientLab Inc., San Francisco, CA, USA) to the W@W-App that is more battery friendly [32]. Fourth, the W@W-App was based on the usability study from a previous version of the W@W-App [21], but did not examine how usable participants found the W@W-App. A user-centred approach may have provided valuable feedback on amendments or adaptations that may be necessary for better implementing occupational mHealth programs to “sit less, move more”. Future research should explore app usability from user experience on mHealth interventions. Finally, this study aimed to provide translational research by gathering data from everyday practice. While this is a strength, it limited sample homogeneity to middle-aged administrative females employed at four Spanish hospitals, which are the most prevalent group of employees in the health administrative sector within the Spanish context. However, the trend of expanding the working life entails a new challenge on forthcoming mHealth occupational interventions [33].

## 5. Conclusions

The W@W-App is a low-cost program that can be applied individually from the organisation level to sedentary-based employees, but feasibility issues such as the battery life of phones should be resolved to prevent high drop-out rates. Whilst no changes in MVPA or sitting patterns were observed during worktime or non-work time on a workday, W@W-App enhanced changes in the number of sitting breaks and time spent in sedentary bouts of a shorter duration on weekends, providing translational evidence that mHealth programs may have the potential to enhance employees’ daily sedentary patterns outside work. Evaluating the impact of occupational “sit less, move more” interventions not only on changes for sitting and PA behaviours during work hours but also outside work would provide a better understating of the impact occupational mHealth programs might have on the overall PA and sedentary patterns.

## Figures and Tables

**Table 1:** Participants’ activity and sedentary behaviour characteristics.

	Average Week	Workday	Working Time	Non-Working Time	Weekends
*n*	132	132	115	115	132
Stepping time (hours)	1.98 (0.50)	2.03 (0.50)	0.71 (0.32)	1.31 (0.41)	1.89 (0.78)
MVPA (minutes)	42.60 (21.55)	47.67 (21.32)	18.38 (11.40)	30.38 (17.19)	33.92 (33.11)
LIPA (hours)	1.28 (0.34)	1.25 (0.35)	0.41 (0.20)	0.83 (0.26)	1.33 (0.51)
Standing time (hours)	5.00 (1.26)	5.04 (1.34)	1.90 (0.93)	3.07 (0.82)	4.95 (1.62)
Sedentary time (hours)	9.50 (1.46)	9.98 (1.72)	4.88 (1.05)	5.20 (1.35)	8.59 (1.99)
Total sedentary bouts (number)	73.85 (25.96)	81.97 (28.36)	41.73 (19.05)	38.59 (15.07)	59.34 (27.20)
<5 min	50.00 (24.65)	55.08 (27.25)	26.77 (17.73)	26.36 (14.51)	40.91 (24.87)
5–10 min	8.96 (2.84)	10.56 (3.54)	6.22 (2.79)	4.35 (1.80)	6.20 (3.41)
10–20 min	7.50 (1.79)	8.66 (2.41)	4.90 (1.73)	3.84 (1.47)	5.39 (2.13)
<20 min	66.47 (26.79)	74.30 (29.52)	37.89 (20.04)	34.56 (15.39)	52.50 (27.78)
>20 min	7.38 (2.03)	7.66 (2.52)	3.83 (1.73)	4.03 (1.51)	6.83 (2.34)
20–30 min	2.92 (1.00)	3.24 (1.27)	1.94 (0.93)	1.37 (0.74)	2.34 (1.28)
30–40 min	1.49 (0.60)	1.60 (0.75)	0.83 (0.60)	0.82 (0.52)	1.25 (0.85)
>40 min	2.98 (1.14)	2.83 (1.32)	1.06 (0.96)	1.84 (0.92)	3.26 (1.49)
40–60 min	1.42 (0.61)	1.45 (0.78)	0.66 (0.60)	0.85 (0.53)	1.38 (0.97)
>60 min	1.55 (0.78)	1.38 (0.88)	0.40 (0.52)	0.99 (0.67)	1.88 (1.03)
>90 min	0.65 (0.47)	124.30 (85.70)	0.08 (0.19)	0.41 (0.41)	0.91 (0.75)
Total sedentary time (minutes)	569.66 (87.66)	599.28 (101.79)	294.43 (64.45)	311.81 (81.69)	518.17 (117.03)
<5 min	62.43 (23.49)	72.04 (28.87)	40.15 (24.69)	30.32 (12.39)	46.00 (23.97)
5–10 min	64.06 (19.87)	75.52 (24.97)	44.39 (19.53)	31.15 (13.08)	44.19 (24.00)
10–20 min	106.40 (25.95)	122.73 (34.71)	69.17 (24.25)	54.85 (21.15)	76.58 (30.56)
<20 min	232.89 (56.03)	270.29 (67.84)	153.71 (53.58)	116.31 (36.81)	166.77 (64.65)
>20 min	336.74 (105.18)	328.94 (120.33)	140.75 (77.76)	198.41 (85.45)	351.40 (131.69)
20–30 min	71.14 (24.48)	78.62 (31.21)	47.04 (22.69)	33.55 (18.18)	57.09 (31.60)
30–40 min	51.00 (20.68)	54.94 (26.12)	28.55 (20.85)	28.18 (18.08)	42.64 (29.14)
>40 min	214.73 (91.28)	195.54 (99.80)	65.26 (63.91)	133.78 (77.45)	251.74 (123.64)
40–60 min	69.53 (29.86)	71.12 (38.61)	32.26 (29.48)	41.72 (26.20)	67.05 (46.92)
>60 min	145.09 (79.87)	124.30 (85.70)	32.87 (45.26)	92.05 (69.94)	184.61 (113.94)
>90 min	78.15 (60.12)	60.42 (61.90)	10.15 (23.66)	50.28 (55.87)	110.93 (101.24)

**Table 2:** Statistically significant outcomes within the intervention group (baseline vs. follow-up) and between groups after 12 weeks.

	Within Intervention Baseline vs. Follow-Up		Between Groups Intervention vs. Comparison	
	Mean Diff. (SD)	*p*	Mean Diff. (95% CI)	*p*
Total week				
MVPA (minutes)	+5.16 (13.28)	0.016		
Working time				
Stepping (hours)	+0.77 (0.22)	0.039		
LIPA (hours)	+0.04 (0.11)	0.031		
Non-working time				
Sedentary breaks of 20–30 min (number)	+0.29 (0.83)	0.037		
Sedentary bouts time of 20–30 min (minutes)	+6.95 (20.37)	0.040		
Weekend				
MVPA (minutes)	+10.89 (21.59)	0.002		
Sedentary breaks of 5–10 min (number)	+1.46 (3.67)	0.015	+2.07 (0.08, 4.07)	0.042
Sedentary bouts time of 5–10 min (minutes)	+10.94 (26.81)	0.013	+14.61 (0.35, 28.87)	0.045
Sedentary bouts time of 10–20 min (minutes)	+13.00 (40.49)	0.046		
Sedentary bouts time of <20 min (minutes)	+29.39 (66.43)	0.007	+35.01 (0.41, 69.62)	0.047

## Supplementary Materials

The following are available online at https://www.mdpi.com/1660-4601/17/23/8844/s1, Table S1: Participants’ activity and sedentary behaviour characteristics, by groups.
