# Supplementary material for: An mHealth Workplace-Based “Sit Less, Move More” Program: Impact on Employees’ Sedentary and Physical Activity Patterns at Work and Away from Work
Source: Int J Environ Res Public Health. 2020 Nov 28;17(23):8844. doi: 10.3390/ijerph17238844 (PMC7730175; doi:10.3390/ijerph17238844)
Supplement: Supplementary file 1 [file ijerph-17-08844-s001.zip › Supplementary material 2.pdf]

walk@work

# FULL D'AUTORITZACIÓ PER A PARTICIPANTS

| Codi Hospital | | Codi participant | | Codi fase estudi | | Codi dispositiu ActivPal | | | | | |
|---|---|---|---|---|---|---|---|---|---|---|---|
| | | | | | | | | | | | |

***Nom***:.........................................................................................................................................

***Data de naixement***:..............................................................................................................

***Departament/Servei/Unitat:***...................................................................................................

***Adreça electrònica professional***:.........................................................................................

***Telèfon professional***:...........................................................................................................

---

He llegit i entenc la informació per a participants relativa a aquesta iniciativa i estic d'acord a participar en Walk@Work, un programa dirigit pel Grup de Recerca en Esport i Activitat Física de la Universitat de Vic-Universitat Central de Catalunya. En el marc de la iniciativa, entenc que se'm demanarà que:

- Segueixi amb la meva rutina habitual a la feina durant una setmana.
- Completi un programa de 12 setmanes de durada amb l'objectiu d'augmentar el temps que camino i reduir el temps que sec a la feina.
- Descarregui l'aplicació Walk@Work al mòbil personal.
- Tingui l'aplicació mòbil activada durant les 12 setmanes de programa i durant la setmana anterior al mateix (un total de 13 setmanes).

També entenc que la iniciativa inclourà:

- Portar el mòbil en una bossa per mesurar el temps assegut i el número de passes caminant durant els dies feiners.
- Registrar les dades que apareixen en l´aplicació mòbil en un diari personal.
- Portar el dispositiu ActivPal durant una setmana abans d´iniciar el programa, una setmana després a la seva finalització i una  setmana als tres mesos d´acabar el programa.
- Accedir a la pàgina Web del programa.
- Emplenar un breu qüestionari sobre l'activitat física general que realitzo, la salut que tinc i el meu benestar.
- Prendre mesures de talla, pes, tensió arterial i circumferència de cintura.

- Enviar comentaris sobre les meves experiències durant la iniciativa. Aquests comentaris podran enregistrar-se amb la meva autorització en cas que vulgui participar en una entrevista.

Entenc que si tinc qualsevol alteració que m'afecti la salut, hauria de consultar amb el metge abans de començar el programa. Puc notar rigidesa muscular o cansament el primer cop que camini, però aquests problemes haurien de remetre a mesura que continuï en el programa. No obstant això, si sento dolor o malestar durant el programa, m'hauria d'aturar i consultar amb el metge.

Participo en l'estudi de manera voluntària. Puc deixar-lo en qualsevol moment, informant l'equip del projecte, però no cal que n'expliqui els motius.

Totes les dades recollides per mitjà d'aquesta iniciativa seran confidencials i només es faran informes de dades resumides i anònimes. Les dades es publicaran de tal manera que no s'utilitzin els noms i que no se'm pugui identificar de cap manera.

He resolt tots els dubtes en relació amb el projecte i estic d'acord a participar-hi.

**Signatura:**.....................................................................................................................................

**Data:**...............................................................................................................................................
