# Supplementary material for: An mHealth Workplace-Based “Sit Less, Move More” Program: Impact on Employees’ Sedentary and Physical Activity Patterns at Work and Away from Work
Source: Int J Environ Res Public Health. 2020 Nov 28;17(23):8844. doi: 10.3390/ijerph17238844 (PMC7730175; doi:10.3390/ijerph17238844)
Supplement: Supplementary file 1 [file ijerph-17-08844-s001.zip › Supplementary material 3.pdf]

walk@work

FORMULARIO DE AUTORIZACIÓN DEL PARTICIPANTE

Código Hospital		Código participante		Código fase estudio		Código ActivPal					

Nombre:...

Fecha de nacimiento...

Departamento/Servicio/Unidad:...

Correo electrónico profesional:..

Teléfono profesional:...

He leído y comprendo la información dirigida al participante que me han suministrado en relación con esta iniciativa y estoy de acuerdo en participar en el programa Walk@Work, un programa dirigido por el Grupo de Investigación en Deporte y Actividad Física de la Universidad de Vic-Universidad Central de Cataluña. Como parte de la iniciativa, entiendo que se me solicitará:

- Que mantenga mi rutina habitual en el trabajo y la pauta de actividad física que sigo normalmente durante 16 semanas.
- Que utilice el aplicativo móvil Walk@Work para conocer el recuento de pasos caminando y de tiempo que estoy sentado en el trabajo durante 16 semanas.
- Que descargue el aplicativo Walk@Work en mi móvil personal.
- Que tenga el aplicativo móvil activado durante las 16 semanas de programa.

También entiendo que la iniciativa implicará:

- Llevar el móvil en una bolsa durante el horario laboral para medir el tiempo sentado y el número de pasos caminando en el trabajo.
- Llevar el dispositivo ActivPal durante una semana en enero 2015, una semana en junio 2015 y otra en septiembre de 2015.
- Completar un breve cuestionario sobre mi actividad física general, mi salud y mi bienestar.
- Tomar medidas de mi talla, peso, tensión arterial y circunferencia de cintura.

walk@work

Entiendo que en setiembre de 2015 tendré la oportunidad de participar en un programa que fomentará “sentarse menos y caminar más mientras trabajo”, la participación en el cuál será totalmente voluntario.

Mi participación en este estudio es voluntaria. Puedo abandonar el proyecto en cualquier momento, informando al equipo del proyecto y sin necesidad de justificar el abandono.

Toda la información recogida mediante esta iniciativa se considerará confidencial y mis datos solo aparecerán en un informe, resumidos y anónimos. Los datos se publicarán sin utilizar mi nombre y de modo que no sea posible identificarme de ninguna manera.

Todas las preguntas que he formulado relativas al proyecto han sido respuestas y acepto participar en este estudio.

Firma:..

Fecha:..
..
