# Supplementary material for: An mHealth Workplace-Based “Sit Less, Move More” Program: Impact on Employees’ Sedentary and Physical Activity Patterns at Work and Away from Work
Source: Int J Environ Res Public Health. 2020 Nov 28;17(23):8844. doi: 10.3390/ijerph17238844 (PMC7730175; doi:10.3390/ijerph17238844)
Supplement: Supplementary file 1 [file ijerph-17-08844-s001.zip › Supplementary material 4.pdf]

walk@work

INFORMACIÓ PER A PARTICIPANTS

Se us ha convidat a participar en una iniciativa saludable al vostre lloc de treball. Abans que decidiu participar-hi, és important que entengueu per què té lloc la iniciativa, què inclou i què espera aconseguir.

Si us plau, llegiu atentament la següent informació i comenteu-la amb altres persones si voleu. Si teniu preguntes o inquietuds, no dubteu a contactar amb l'equip de Walk@Work del vostre hospital (Roser Bausà i Lidia Navarro).

De què tracta la iniciativa?

Estar assegut durant períodes llargs de temps és nociu per a la salut. Reduir el temps assegut en menys de 7 hores al dia millora la salut cardiovascular i redueix el risc de desenvolupar diabetis, sobrepès o obesitat; independentment de si es fa exercici físic o no en el temps de lleure. El fet de practicar exercici físic no elimina els riscos per a la salut de seure durant temps perllongats.

Malgrat aquests beneficis, les oportunitats de poder fer activitat física es veuen reduïdes per una sèrie de barreres, entre les quals s'inclouen les ocupacions sedentàries.

Ens agradaria donar-vos oportunitats de seure menys i caminar més durant la vostra atrafegada jornada laboral. Volem millorar el vostre benestar mentre treballeu tot gaudint d´una feina més activa de forma sostenible. Per això, hem dissenyat un programa basat en tecnologia mòbil i Web, al qual ens agradaria que accedíssiu i que voldríem que utilitzéssiu.

Què hauré de fer en l'estudi?

Conèixer el temps que normalment passeu asseguts a la cadira i el número de passes que feu caminant.

Mesurarem minut a minut el temps que passeu asseguts, dempeus o caminant objectivament, mitjançant un petit dispositiu ActivPal que es col·loca a la cama durant 7 dies. Prendrem aquesta mesura al gener, juny i a l´octubre de 2015 per conèixer el vostre patró habitual d´activitat física i com aquest es manté al llarg de l´any. Això ens permetrà donar-vos un informe detallat que explicarà el vostre patró d´activitat física habitual a finals de novembre de 2015.

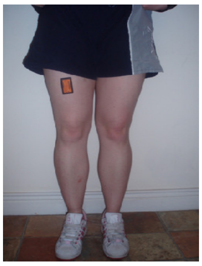

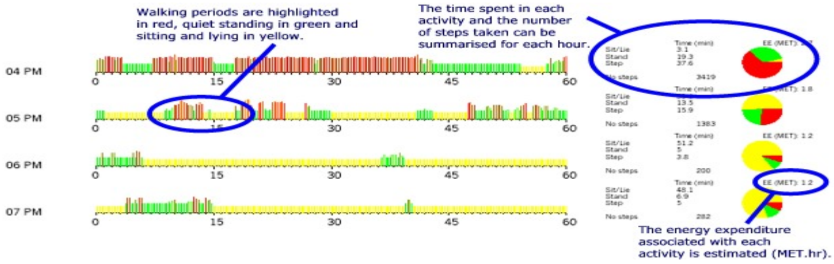

Descarregar l'aplicació Walk@Work al mòbil personal *(Smartphone tipus Android versió 4.1 o superiors)* i mantenir-lo activat durant 13 setmanes

Us permetrà prendre consciència *"in situ"* del temps que esteu asseguts a la cadira i el temps que camineu diàriament a la feina. Tot treballant, tindreu accés i observareu a temps real el temps que acumuleu estant assegut, el nombre de passes que feu, el nombre de vegades que us aixequeu i el temps que fa que no us moveu de la cadira.

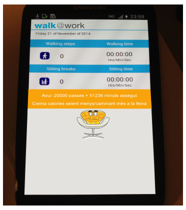

Tindreu una bona idea de com evoluciona el "dia a dia" del temps que romaneu asseguts i el temps que camineu durant el vostre horari laboral (23 de febrer – 24 de maig 2015). L´aplicació enviarà aquestes dades a la vostra part privada de la Web (walkatwork.uvic.cat/es) la qual farà un petit anàlisi i us retornarà missatges de feedback. Us informarà sobre la vostra conducta habitual d´activitat física a la feina i com aquesta afecta el vostre estat de salut.

Col·locar el mòbil en un bossa petita que s´enganxa al cinturó o a un cinturó addicional durant les hores de feina

Aquesta posició de mòbil és la que permet obtenir mesures reals, objectives i fiables del temps que acumuleu assegut i del nombre de passes que feu. La bossa us permet continuar amb l´ús habitual del mòbil.

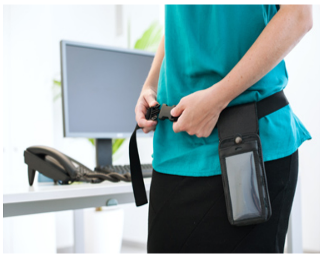

Al final de les 13 setmanes us podreu quedar amb la bossa i si ho desitgeu, podreu continuar utilizant l´aplicació mòbil per seguir controlant la quantitat de passos i el temps que romaneu asseguts durant la jornada laboral.

Establir la línia de base per conèixer el temps que passeu a la cadira i el temps que camineu

Abans d´accedir al programa “Walk@Work” (2 de març 2015), voldríem fer-nos una idea sobre com és el vostre dia laborable típic. Així que se us demanarà que continueu seient i caminant com ho feu normalment durant una setmana (23-28 de febrer ambdós inclosos), cosa que constitueix la línia de base.

L’aplicació mòbil enviarà les dades directament a la part privada de la web. Utilitzant aquesta informació, la pàgina web establirà les vostres metes personalitzades, permetrà fer-ne un seguiment i també fer-vos comentaris sobre els canvis que aniran succeint en la quantitat de temps que seieu i que camineu al llarg de dotze setmanes.

Anotar el número de passes que passeu caminant, el temps que passeu assegut i el número de vegades que us aixequeu de la cadira durant la jornada laboral.

Se us donarà un diari perquè anoteu tota aquesta informació durant els últims cinc minuts abans d´acabar la jornada laboral. Es tracta de registrar les mesures que ha comptabilitzat la vostra aplicació mòbil. No necessiteu més d´un parell de minuts per fer-ho.

Accedir a la pàgina web de Walk@Work durant 12 setmanes (2 març – 24 maig)

Se us donarà un accés individual a un programa web interactiu i confidencial que, a través d’una sèrie de funcions i estratègies, té com a objectiu ajudar-vos a disminuir el temps que seieu per mitjà d’un increment del temps que camineu (walkatwork.uvic.cat/es).

Aplicareu estratègies individualitzades que s’integren i complementen amb les tasques i responsabilitats pròpies del vostre lloc de treball. Decidireu quines són les estratègies més adients i el millor moment del dia per aplicar-les, sense interferir en la vostra rutina laboral. Podreu seguir els vostres progressos mitjançant gràfiques i podreu compartir les vostres experiències en el blog o twitter del programa. Mitjançant autorització, també podreu seguir les fites assolides per altres participants.

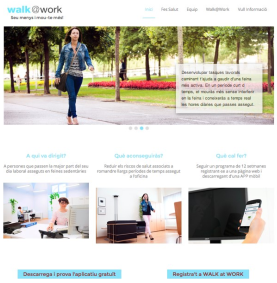

Altres mesures

Walk@Work té l'objectiu de proporcionar guiatge, consells i suport a mida per fer canvis sostenibles en la quantitat de temps que seieu i esteu en moviment a la feina. Ens agradaria saber com aquests canvis sostinguts influeixen en el vostre estat de salut. Per aquest motiu, també mesurarem la vostra talla, pes, tensió arterial, circumferència de cintura i us podem fer preguntes sobre el vostre benestar.

També ens interessen molt els vostres comentaris sobre la iniciativa. Per això en alguns moments podem demanar-vos que participeu en discussions de grup en les quals comentareu les vostres experiències sobre Walk@Work. Aquesta activitat és discrecional i us demanarem autorització per enregistrar els comentaris que feu.

Què passa si no puc participar en la iniciativa sencera?

Cap problema, així i tot hi podeu participar. Entenem que la feina i altres compromisos poden canviar al llarg del temps. Si hi ha setmanes que no sou a la feina o hi ha una setmana laboral no habitual, podeu indicar-ho en un full de registre d´incidències.

Quins són els avantatges i els desavantatges de participar-hi?

No hi hauria d'haver desavantatges llevat de perdre una mica de temps familiaritzant-se amb l'aplicació mòbil i la pàgina web. També haureu d'interactuar amb la pàgina web i portar el mòbil dins la bossa. No obstant, tot ha estat dissenyat i testejat en un estudi d´usabilitat per a que el programa interferixi el menys possible en la vostra rutina habitual.

Participar-hi té diversos avantatges. Prendreu part d'una iniciativa que pot suposar-vos beneficis sobre la salut i benestar, l'estil de vida i la manera com us sentiu a la feina. Us farem saber de manera confidencial els vostres progressos i, si voleu, us donarem guiatge i consells sobre el que signifiquen.

Com s'utilitzarà la meva informació?

La vostra informació s'utilitzarà per establir els vostres objectius i fer un seguiment del vostre progrés al llarg del programa. La utilitzarem per avaluar l'impacte que la iniciativa té en l'activitat física, en el temps que se seu i en el benestar durant la jornada laboral. Després d´aquesta avaluació, esperem poder estendre aquesta iniciativa a diferents llocs de treball de Catalunya i l´Estat Espanyol.

Codi ètic

Participar en la iniciativa és completament voluntari. La podeu deixar en qualsevol moment sense haver-ne d'explicar els motius. Se us demanarà signar un document de consentiment informat.

Tota la informació serà confidencial i només es faran informes de dades resumides anònimes. Les dades recollides es publicaran de manera que no aparegui el vostre nom i que no se us pugui identificar.

El Comitè d´Ètica del Hospital de la Santa Creu i Sant Pau de ha autoritzat aquest estudi. Podeu discutir la vostra participació en aquest estudi amb el personal del projecte escrivint un correu electrònic o trucant a la Roser Bausà o Lidia Navarro.

Què he de fer a continuació?

Si us plau, penseu detingudament si voleu participar en la iniciativa. Si voleu participar-hi, poseu-vos en contacte amb la Roser Bausà o Lidia Navarro durant la propera setmana.

Esperem participar plegats en aquesta iniciativa!

Cordialment, *L´equip de Walk@Work*
