# Supplementary material for: An mHealth Workplace-Based “Sit Less, Move More” Program: Impact on Employees’ Sedentary and Physical Activity Patterns at Work and Away from Work
Source: Int J Environ Res Public Health. 2020 Nov 28;17(23):8844. doi: 10.3390/ijerph17238844 (PMC7730175; doi:10.3390/ijerph17238844)
Supplement: Supplementary file 1 [file ijerph-17-08844-s001.zip › Supplementary material 5.pdf]

walk@work

INFORMACIÓN PARA EL PARTICIPANTE

Le invitamos a participar en una iniciativa saludable en su puesto de trabajo. Antes de que decida participar, es importante que comprenda las razones de la iniciativa, sus implicaciones y sus objetivos.

Tómese un tiempo para leer esta información atentamente y coméntela con otras personas si lo desea. Si tiene alguna duda o preocupación, comuníquese con el equipo de «Walk@Work» en su hospital (nombre, apellidos y correo electrónico).

¿En qué consiste la iniciativa?

Estar sentado durante periodos de tiempo largos es nocivo para la salud. Reducir el tiempo sentado en menos de 7 horas al día mejora la salud cardiovascular y reduce el riesgo de desarrollar diabetes, sobrepeso u obesidad; independientemente de si realiza o no ejercicio físico en el tiempo de ocio. Hacer ejercicio físico durante el tiempo libre no elimina los riesgos para la salud de permanecer sentado durante periodos de tiempo prolongados.

A pesar de los beneficios, son diversos los obstáculos, entre los cuales los trabajos sedentarios, que impiden poder hacer actividad física diariamente. Queremos brindarle la oportunidad de permanecer menos tiempo en la silla y caminar más durante su ajetreada jornada laboral. Queremos que se sienta mejor disfrutando de un trabajo más activo de forma sostenible.

Por este motivo, querríamos evaluar su conducta habitual de actividad física y el impacto que tiene sobre su estado de salud. Mediremos el tiempo diario que acumula estando sentado y el número de pasos que da caminando. Posteriormente, le aconsejaremos e informaremos en setiembre de 2015. Esto les proporcionará información valiosa sobre varios aspectos de su salud, incluyendo la cantidad de actividad física que hace normalmente y la interpretación de la misma.

En enero de 2016 tendrá la oportunidad de empezar un programa de sentarse menos y caminar más en el trabajo durante 12 semanas. Si decide participar en el programa, partiremos de los datos obtenidos en esta primera fase para fijar sus objetivos relativos a reducir el tiempo que permanece sentado, aumentar el número de pasos caminando y hacer un seguimiento de su progresión.

¿Qué deberé hacer en el estudio?

Medir el tiempo que suele pasar sentado en la silla y el número de pasos caminando.

Mediremos minuto a minuto el tiempo que pasa sentado, de pie o caminando objetivamente, mediante un pequeño dispositivo ActivPal que se coloca en la pierna durante 7 días. Tomaremos una medición en enero, junio y otra en octubre de 2015 para conocer su patrón habitual de actividad física y como se mantiene a lo largo del año. Esto nos permitirá darle un informe detallado que explicará su patrón de actividad física habitual a finales de noviembre de 2015.

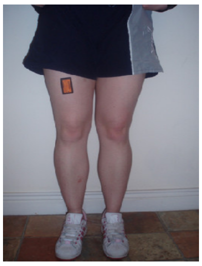

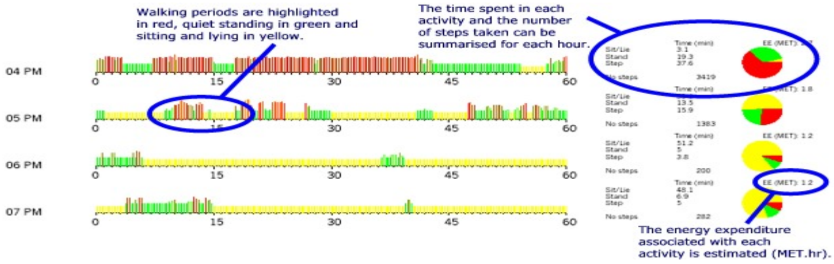

Descargar la aplicación Walk@Work en su teléfono móvil *(Smartphone tipo Android versiones 4.1 o superiores)* y mantenerlo en funcionamiento durante 16 semanas

Le permitirá tomar conciencia *"in situ"* del tiempo que está sentado/a en la silla y el tiempo que camina diariamente en el trabajo. Mientras trabaja, tendrá acceso y observará en tiempo real los minutos diarios que acumula estando sentado, el número de pasos que da caminando, el número de veces que se levanta y el tiempo que hace que no se mueve de la silla.

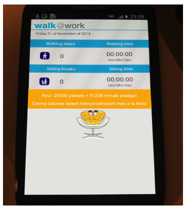

Deseamos darle una idea sobre la evolución "día a día" del tiempo que permanece en la silla y el tiempo que camina durante su horario laboral (16 febrero - 8 junio 2015). A partir del mes de julio, analizaremos la información recibida de su aplicación móvil - la cual se guarda en una base de datos confidencial - para profundizar en el estudio de su conducta habitual diaria de caminar y sedentarismo (tiempo sentado/a) en el trabajo. Podremos informarle sobre sus resultados e implicaciones para su salud a finales de noviembre de 2015.

Colocar el teléfono móvil en una pequeña bolsa que se acopla a su cinturón o un cinturón adicional durante su horario laboral.

Esta posición del móvil es la que permite obtener medidas reales, objetivas y fiables del tiempo diario que acumula estando sentado y el número de pasos que da caminando. La bolsa le permite continuar con el uso habitual del móvil.

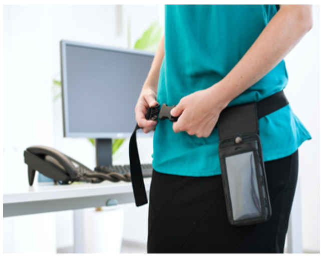

Al final de las 16 semanas, podrá quedarse con la bolsa y si lo desea, podrá continuar utilizando la aplicación móvil para seguir controlando la cantidad de pasos y el tiempo que permanece sentado durante la jornada laboral.

Otras medidas

El programa pretende guiarle, aconsejarle y apoyarle de modo personalizado para identificar como sus hábitos de actividad física influyen en su salud. Por este motivo, estamos muy interesados en evaluar bienestar, talla, peso, tensión arterial y circunferencia de cintura para darle feedback sobre el impacto de su conducta de actividad física y tiempo sentado/a en su estado de salud. Solicitaremos su permiso para registrar estos datos.

Si lo desea, la información obtenida servirá para establecer sus objetivos relativos a sentarse menos y caminar más en el programa “Walk@Work” que empezará en enero de 2016. En setiembre-octubre-noviembre de 2015 el equipo de Walk@Work se pondrá en contacto con usted para preguntarle si desea participar en el programa.

¿Qué sucede si no puedo participar en toda la iniciativa?

No se preocupe. Sigue pudiendo participar. Somos conscientes de que el trabajo y otros compromisos varían con el tiempo. Si durante algunas semanas usted debe desplazarse de su puesto de trabajo o tiene lugar alguna semana laboral especial, usted puede anotarlo en una hoja de registro de incidencias.

¿Cuáles son los beneficios e inconvenientes de participar?

A parte de tener que pasar un poco de tiempo familiarizándose con la aplicación móvil y llevar el dispositivo ActivPal, no debería haber ningún tipo de inconveniente. Durante el programa deberá tener activado la aplicación y llevar el móvil en la bolsa. No obstante, todo ha estado diseñado y probado en un estudio de usabilidad para que interfiera el mínimo posible en su rutina habitual.

Los beneficios son diversos. Participará en una iniciativa que puede beneficiar su salud, bienestar, estilo de vida y sensaciones en el trabajo. En confianza, le informaremos sobre sus resultados y, si lo desea, le guiaremos y aconsejaremos sobre lo que significan.

¿Qué uso se hará de mi información?

Su información se utilizará para identificar su conducta habitual de actividad física y el impacto que tiene sobre su estado de salud. Si en setiembre-octubre de 2015 decide participar en el programa de sentarse menos y caminar más en el trabajo (Walk@Work), también se utilizará para establecer sus objetivos iniciales de caminar, sentarse menos y hacer un seguimiento de su progreso a lo largo del programa.

También la utilizaremos para evaluar el impacto de la iniciativa de actividad física en horario laboral sobre el bienestar. Esperamos poder extender dicha iniciativa en diferentes centros de trabajo del Estado Español.

Ética

Su participación en la iniciativa es completamente voluntaria. Puede abandonarla en cualquier momento sin necesidad de justificar su abandono. Se le pedirá que firme un documento de consetimiento informado.

Toda la información será confidencial y sólo se presentaran datos anónimos en un resumen. Los datos se publicarán de modo que su nombre no se utilice y que a usted no se le pueda identificar.

Este estudio ha sido revisado por el Comité de Ética de XXXXXXXXX. Puede discutir su participación con el equipo de Walk@Work llamando a XXXXXXX o enviando un correo electrónico a XXXXXXX.

¿Qué sucede a continuación?

Piense detenidamente sobre si desea participar en la iniciativa. Si alguna cosa no le parece clara o precisa mayor información, contacte con nosotros. Si desea tomar parte, póngase en contacto con XXXXXXXX (correo electrónico) durante la próxima semana.

Esperamos poder participar en esta iniciativa con usted!

Atentamente,

El equipo Walk @ Work
